# Effect of Duyun Compound Green Tea on Gut Microbiota Diversity in High-Fat-Diet-Induced Mice Revealed by Illumina High-Throughput Sequencing

**DOI:** 10.1155/2021/8832554

**Published:** 2021-02-09

**Authors:** Caibi Zhou, Xiaolu Zhou, Zhirui Wen, Zaibo Yang, Ren Mu, Yuyan Song, Xin Mei, Dylan O'Neill Rothenberg

**Affiliations:** ^1^College of Biological Science and Agriculture, Qiannan Normal University for Nationalities, Duyun 558000, China; ^2^College of Horticulture and Landscape Architecture, Hunan Agricultural University, Changsha 410000, China; ^3^College of Horticulture, South China Agricultural University, Guangzhou 510642, China

## Abstract

Intake of a high-fat diet (HFD) is closely related to disorders of the intestinal microbiota, which plays a key role in the pathogenesis of obesity. Duyun compound green tea, an ancient Chinese drink, is widely consumed to reduce weight, although the mechanism is not clear. In this study, 50 mice were randomly divided into 5 groups: normal control group (CK), HFD model control group (NK), positive control group with medicine (YK), low-dose compound tea group (DL), and high-dose compound tea group (DH). After 4 weeks of intervention, the feces of mice were taken under sterile conditions and evaluated using Illumina high-throughput sequencing technology. The results showed that the diversity of intestinal microbiota was the highest in the CK group, the lowest in the NK group, and relatively increased in the compound tea treatment group. Second, there were differences in intestinal microbiota in each group, among which the beneficial bacteria in the intestinal tract of the CK group were higher than those in the other groups, while the beneficial bacteria in each compound tea treatment group were more abundant than those in the NK group, in which harmful bacteria in the intestinal tract were found to be the highest. These results suggest that compounds in tea may be able to attenuate imbalances of intestinal microbiota induced by poor diet, acting as a therapeutic agent in obesity or other diseases associated with gut dysbiosis.

## 1. Introduction

Intestinal microbiota function in a symbiotic microbial ecosystem with the human body, affecting multiple systems in the host, particularly digestion and absorption of food. Regardless of whether the host is in a state of health or disease, multiple physiological and metabolic characteristics are inevitably affected by the gut microbial network [1]. Improvements to the overall intestinal microbial status can be obtained through means such as reducing harmful bacteria such as *Enterobacteriaceae*, activating certain host genes, regulating metabolism, increasing intestinal butyrate and other short-chain fatty acids (SCFA), reducing abnormal production and absorption of propionate, and reducing the accumulation of protein fermentation products such as hydrogen sulfide and ammonia [2]. These improvements can effectively regulate appetite, aid in food digestion, promote intestinal immunity, increase insulin resistance [3], increase resistance to pathogenic bacteria, train the immune system, strengthen cancer treatment [4–8], mitigate fatty liver, reduce hyperlipidemia, and attenuate metabolic disorder [9–11].

Tea is rich in tea polyphenols, amino acids, tea polysaccharides, and other substances exhibiting characteristics such as antioxidant, antitumor, antiradiation, hypoglycemic, hypolipidemic, probiotic, and other health effects [12–16]. Intestinal microorganisms can transform tea polyphenols and other components into short-chain fatty acids, *γ*-pentalactone, phenolic acids, and other metabolites, which can effectively improve insulin sensitivity, adiponectin levels, total cholesterol, and triglyceride levels, and prevent the accumulation of fat in the liver [17], thereby improving parameters related to obesity regulation. Furthermore, tea can regulate the intestinal microbial structure, indirectly participating in host energy absorption and food metabolism, gene expression, and immune response [18]. Additionally, tea polyphenols and other bioactive substances in green tea can reduce the fasting blood glucose level and mesenteric fat and increase the serum insulin level, which may work to lower blood lipid content by preventing *β* cell damage and changing the bacterial community in the intestine [19].

Obesity and related metabolic disorders have been suggested to be a major global public health threat [20] and are closely related to disorders of the intestinal microbiome [21], such that changes in composition or activity of intestinal microbiota play a key role in the pathogenesis of obesity and related diseases [22]. Diet is a key factor in shaping the structure of intestinal microbiota [23, 24]. In particular, a weight-loss mechanism of natural products has been shown to be interrelated with the regulation of intestinal microbiota. The regulation of intestinal microbiota through plant diet intervention is generally considered an effective strategy in obesity prevention. Currently, many foods and drugs that regulate intestinal microbiota have been discovered. However, targeted regulation by drugs remains a topic of investigation. Increasing attention in modern biomedical research has focused on the prevention of metabolic diseases through the regulation of gut microbial imbalance, *via* alterations of the intestinal microbial community. Duyun compound green tea is a traditional folk formula composed of green tea and five other Chinese herbal medicines, which are broadleaf holly, sweet tea, lotus, wild mint, and honeysuckle. They can ameliorate intestinal conditions [25–27] or antiobesity [28, 29]. In this study, it was used to change the composition of intestinal microbiota, providing a new treatment option for metabolic syndrome and providing new methods and new ideas for the development of personalized medicine and obesity treatment.

Currently, there are many studies on the functional components of tea; however, studies on the regulation of intestinal microbiota diversity by compound tea have rarely been reported. The ancient Duyun compound tea has been used to reduce weight, but there is no study on its mechanism. In this paper, Illumina high-throughput sequencing technology was used to study the effect of Duyun compound green tea consumption on the intestinal microbiota diversity of high-fat-diet (HFD)-induced mice. The purpose of this study was to explore the preventive and modulatory effects of Duyun green tea compounds on intestinal microbiota-related diseases and provide references for the mechanisms by which compound tea is able to regulate intestinal microbiota.

## 2. Materials and Methods

### 2.1. Materials and Reagents

The compound tea materials utilized for research consist of 30% Duyun ancient tea (*Camellia sinensis*) leaves, 14% Duyun broadleaf Holly (*llex latifolia* Thunb.), 14% Duyun original tree sweet tea (*Rubus suavissimus*), 14% leaf of lotus (*Nelumbo* SP.), 14% wild mint (*Menthae Haplocalycis Herba*), and 14% honeysuckle (*Lonicerae japonicae flos*), which were purchased from Guizhou Bishu Technology Service Co., Ltd. (Guizhou, China). Xuezhikang capsules were provided by Beijing Beida Weixin Biotechnology Co., Ltd. (Beijing, China). SPF (specific pathogen free) male Kunming mice were provided by Hunan SJA Laboratory Animal Co., Ltd. (Hunan, China). The high-fat diet was composed of the following ingredients: 1.5% cholesterol, 10.0% lard, 5.0% yolk powder, 0.5% bile salt, and 83.0% conventional feed.

### 2.2. Sample Preparation

Tea leaves were infused in boiling water with the ratio of 1 : 10 for 30 minutes; then filtered with two layers of industrial gauze when the tea had cooled slightly. The tea dregs were extracted again for 20 minutes with the ratio of 1 : 8 using vacuum filtration. Two filtrates were mixed and brought to a certain concentration using a rotary evaporator (R201B; Shanghai Shensheng Technology Co., Ltd.), precooled at −80°C for 12 h, vacuum freeze-dried for 28 h, collected as dry powder, then sealed, and stored in a −80°C refrigerator until further use. The extract compounds from the tea leaves were listed in the supplemental data (Table S1).

### 2.3. Animal Experiments

Fifty 5-week-old male KM mice of SPF grade were housed in a room with a temperature range of 20∼26°C and relative humidity of 50%∼60%. After 7 days of adaptive feeding, they were randomly divided into 5 groups, with 10 mice in a single cage for each group. Normal group (CK) was fed normal diet and purified water; model group (NK) was fed a high-fat diet (HFD) and purified water; positive group (YK) was fed a HFD along with a lipid-lowering medication called “Xuezhikang” at a dose of 90 mg·kg^−1^·d^−1^; low-dose group (DL) was fed a HFD and 210 mg·kg^−1^·d^−1^ of compound green tea extract; and high-dose group (DH) was fed a HFD and 840 mg·kg^−1^·d^−1^ of compound green tea extract. The doses were chosen based on safety evaluation in preliminary experiment. The medicine and compound green tea extract were fed by gavage to mice. All the food and water were sterile. During the experiment, the activity of mice was observed and body weights were recorded. At the end of the 28-day test, the feces of each mouse were collected aseptically and independently and put into a 1.5 mL sterilized centrifuge tube, labeled, and frozen at −80°C for standby.

### 2.4. Genomic DNA Extraction and PCR Amplification of Fecal Microbiota

One hundred milligram stool samples with 1.4 mL stool sample lysis buffer (ASL) were prepared after centrifugation. The total DNA of the sample microorganisms was extracted using the GENEWIZ fecal DNA extraction kit, and the detailed procedure was carried out in accordance with the instructions.

Using 30–50 ng DNA as a template, V3 and V4 regions were amplified with the upstream primer including “CCTACGGGRRBGCASCAGKVRVAAT” sequence and the downstream primer including “GGACTACNVGGGTWTCTAAATCC” sequence. Additionally, the PCR product end of 16S rDNA was added with the connector with index by PCR for sequencing by the next generation sequencing [30]. The raw data have been deposited in the NCBI Sequence Read Archive database with the BioProject accession number PRJNA633980.

### 2.5. Bioinformatics Analysis

The quality of the library was detected using the Agilent 2100 Bioanalyzer (Agilent Technologies, Palo Alto, CA, USA), and the library concentration was detected using a qubit2.0 fluorometer (Invitrogen, Carlsbad, CA). After the DNA library was mixed, PE250 double-terminal sequencing was carried out according to the instruction manual of Illumina MiSeq (Illumina, San Diego, CA, USA), and the sequence information was read by MiSeq's own MiSeq control software (MCS). Bioinformatics analysis was performed on the GENEWIZ platform (https://www.genewiz.com.cn/). Briefly, OTUs (operational taxonomic units) and their diversities were recognized by Qiime (1.9.1) and Vsearch (1.9.6); PCoA (principal co-ordinates analysis) and other figures were drawn by R (3.6.3). LDA (linear discriminant analysis) effect size was calculated on LEfSE Online tools.

## 3. Results

### 3.1. Sample Sequencing and OTU Cluster Analysis

High-fat diet induced the weight gain of mice (NK group), and high dose of compound tea (DH group) could restored it as the Xuezhikang (YK) did (Table S2 in supplemental data). After high-throughput sequencing based on the Illumina MiSeq platform, a total of 911,515 effective 16S rRNA gene sequences (raw reads) were obtained from 15 samples in 5 groups. After screening, filtering, and optimization, a total of 747,339 high-quality sequences, 2,858 OTUs, with an average of 49,882 sequences, and 190 OTUs for each sample, were obtained after further quality control of the spliced sequences, such as the removal of mosaics.

There were 126,533 sequences and 686 OTUs in the CK group, 138,458 sequences and 568 OTUs in the DH group, 167,129 sequences and 550 OTUs in the DL group, 149,518 sequences and 546 OTUs in the NK group, and 165,701 sequences and 580 OTUs in the YK group. The samples and related information of each group are shown in Table 1.

**Figure 1:**
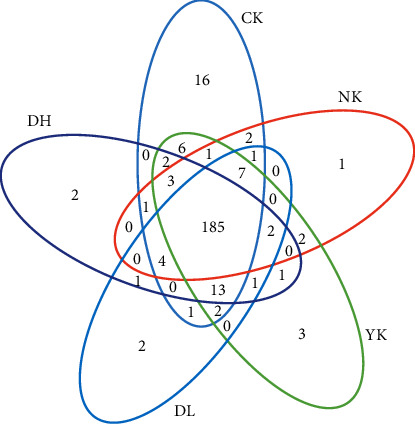
OTUs' Venn diagram. Each petal represents a group, the number on the petal represents the number of species unique to the group, and the middle circle represents the number of species shared by all groups.

The number of unique and shared OTUs between each group of mice was analyzed by OTU's Venn diagram to compare the degree of microbial composition overlap and similarity between the individual components. There were 185 species in 5 groups, including 16 unique species in CK, 2 unique species in DH, 2 unique species in DL, 3 unique species in YK, and 1 unique species in NK. The CK group had the most abundant species, which was higher than other groups (Figure 1).

**Figure 2:**
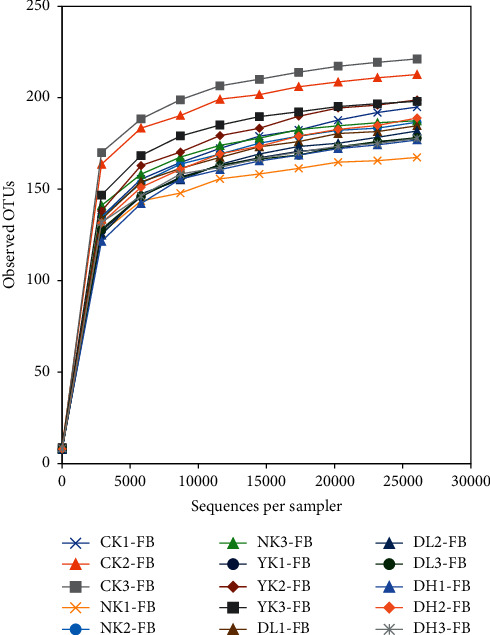
Rarefaction curves in the figure show the number of sequences per sample and the ordinate is the number of OTUs. The results showed that the increasing speed and trend of new species were observed with the increase of sequencing depth.

Dilution curves are widely used to judge whether the sample size is sufficient and can estimate species richness. It refers to a curve constructed between a certain number of individuals randomly selected from the sample and the number of species it can represent. When the curve tends to be flat, the number of samples is reasonable. On the basis of obtaining 97% similarity of OTU in this experiment, we further plotted the dilution curve to determine whether the amount of data for sequencing was sufficient. According to Figure 2, the sample size used in this study is 15. With the deepening of sequencing data, the number of OTUs increased significantly, the increasing trend of OTUs in each group did not change significantly but gradually tended to be slow. This shows that although the amount of sequencing data continued to increase, the number of OTUs obtained was also limited. Therefore, the amount of data we sequenced was sufficient to cover all species in the sample, indicating that the amount of sequencing data was sufficient for further analysis.

### 3.2. Alpha Diversity Analysis of Intestinal Microbial Community

**Figure 3:**
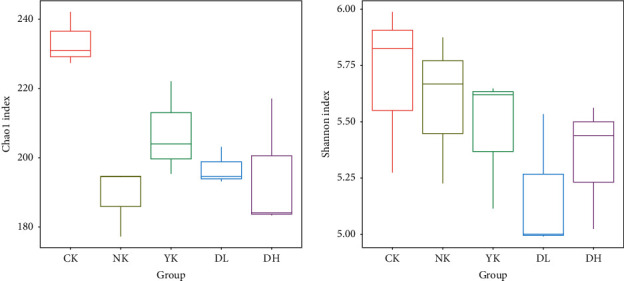
Chao1 index and Shannon index indicate species diversity of the samples. The box chart represents the minimum value, the lower quartile, the median, the upper quartile, and the maximum value from the bottom to the top.

The species diversity of the microbial community is usually expressed by the richness index and diversity index. The larger the value is, the greater the richness will be, while the Shannon index increases with greater species diversity. By analyzing the alpha diversity index, we can measure the species diversity of the samples and draw the box graph of each sample's Chao1 index and Shannon index (Figure 3). Compared with the CK group, the number of OTUs in the other four groups was significantly less. The fecal microbiota richness of tea treatment group indicated that tea was able to significantly change the fecal microbiota richness of mice.

### 3.3. Principal Coordinate Analysis of Intestinal Microbial Community

**Figure 4:**
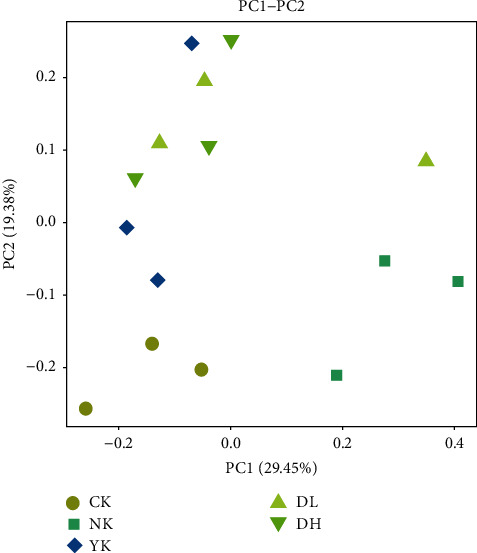
PCoA diagram of samples at the genus level of intestinal microbiota.

Through PCoA analysis (Figure 4), we could see that the samples of the CK group were distinguishable from the other four groups, indicating that the species composition of the non-HFD group was quite different from the other four groups. For the CK and NK groups, the mice samples gathered to form an independent area, and the difference between the groups was greater than differences within the group. For the samples of DL, DH, and YK groups, the samples in each group could not be completely gathered to form an independent area based on sampling difference, but there was a significant distance from the NK group. These data suggest that consumption of Xuezhikang medicine and both doses of tea were able to attenuate HFD-induced alterations in intestinal microbial communities; however, the effect of the three treatments was not entirely distinguishable from one another.

### 3.4. Cluster Analysis of Intestinal Microbial Community by UPGMA Tree

**Figure 5:**
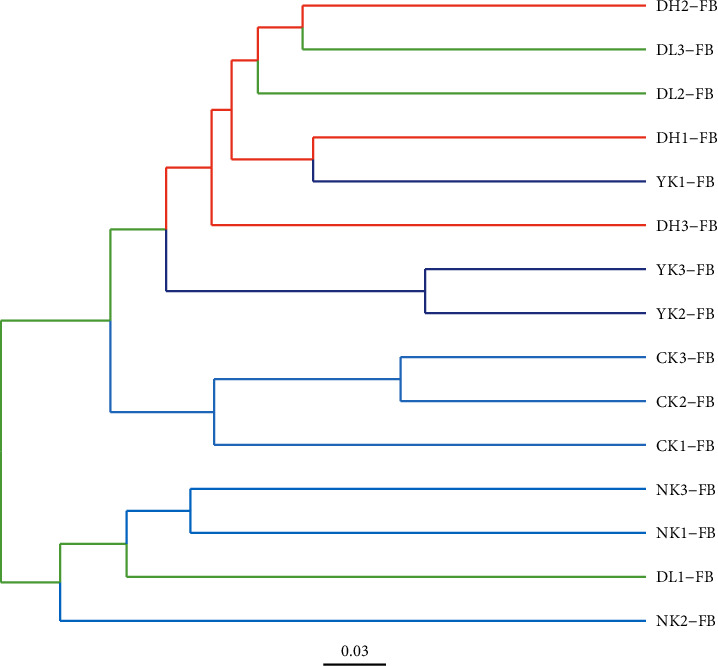
UPGMA tree diagram of samples.

Through UPGMA tree cluster analysis (Figure 5), it was clear that mice in the CK group were significantly different than mice in all HFD treatment groups. Furthermore, three groupings, those of the CK, NK, and YK/DH groups, all maintained their own unique cluster branches, indicating that there were significant differences among them. The YK and DH groups overlapped with each other, however, did not overlap with the CK and NK groups, while the DL group overlapped with YK/DH in addition to the NK group, suggesting that the low-dose tea group was less effective attenuator of HFD-induced microbial alterations than YK and high-dose tea treatments.

### 3.5. The Structure of Microbial Community and Abundance Changes

**Figure 6:**
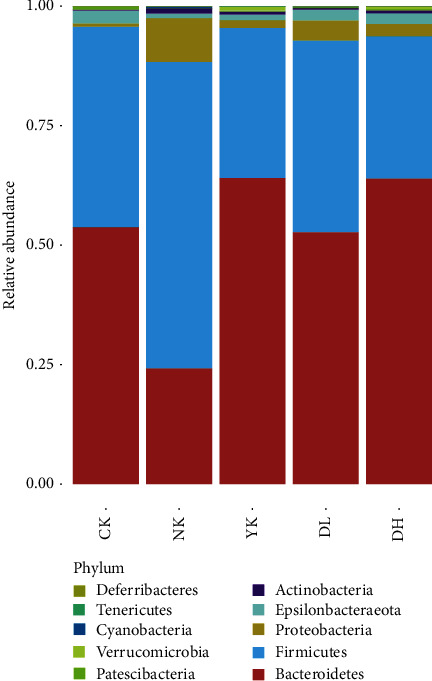
Structure of the microbial community and abundance changes at phylum level. The abscissa in the figure is the sample component, and the ordinate represents the sample richness. Different bacterial groups are represented by different colors.

According to the histogram of microbial community composition at the phylum level, it was found that *Firmicutes* and *Bacteroidetes* were the main dominant phyla of fecal microbiota in all mice, although other important phyla such as *actinomycetes*, *proteus*, soft wall phylum, and *verruca* were also detected. The results of comparative analysis of *Firmicutes* and *Bacteroidetes* in each group are shown in Figure 6. In the NK group, the content of *Firmicutes* increased, *Bacteroidetes* decreased, *Proteobacteria* increased, and epsilon bacteria decreased compared with all other groups, indicating that HFD-induced obesity caused a change in intestinal microbiota composition. Following low-dose tea treatment, *Firmicutes* and *Bacteroidetes* remained the dominant phyla, and the *Firmicutes*/*Bacteroidetes* ratio was attenuated to a level insignificantly different from that of the CK group, indicating that low-dose tea treatment could effectively regulate intestinal microbiota communities, restoring microbial imbalances induced by a high-fat diet to a level similar to a non-HFD group. Both the DH and YK groups showed attenuations in the *Firmicutes*/*Bacteroidetes* ratio at a level beyond that of the CK group, suggesting not only a reversal of harmful imbalances brought about by HFD but also a net improvement to microbial communities beyond the status quo of HFD-aversion. The microbial community compositions at other categorical levels were shown in supplemental data (Figures S1–S5).

**Figure 7:**
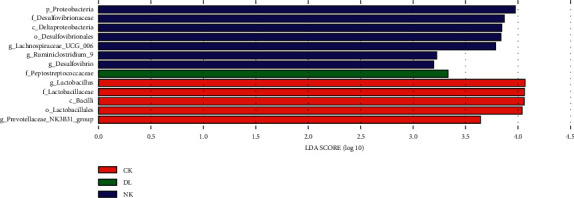
LEfSE diagram of the significant taxonomies between groups. The vertical coordinate is the species groups with significant differences between groups, and the horizontal coordinate is the bar graph to show the LDA difference analysis of each species group. The scores (LDA value is greater than 3) are sorted according to the scores, so as to describe their differences in different groups of samples. The longer the length is, the more significant the difference will be. The different colors of the bar chart indicate the sample groups with higher abundance.

At the species level, the LDA difference analysis of fecal microbiota in CK, NK, YK, DL, and DH mice was compared to the strains with scores greater than 3. The column distribution of LDA values is shown in Figure 7. The cladogram could be found in supplemental data (Figure S6). Only dominant species with significant difference were found in the CK, NK, and DL groups, and the numbers of dominant species with significant difference were 5, 7, and 1, respectively. The dominant species with significant difference in CK were *Bacilli*, *Lactobacillales*, *Lactobacillaceae*, *Lactobacillus*, and *Prevotellaceae*; the dominant species with significant difference in NK were *Proteobacteria*, *Deltaproteobacteria*, *Desulfovibrionaceae*, *Lachnospiraceae*, *Ruminiclostridium*, and *Desulfovibrio*; the dominant species with significant difference in DL was *Proteobacteria*.

## 4. Discussion

Intestinal microbiota are able to form symbiotic relationships with the host and play a key role in host homeostasis. In turn, homeostatic stability has an important impact on key physiological functions of the body. In this study, the microbial community and structure in the feces of mice were detected by the high-throughput sequencing technology of Illumina. It was found that there were some differences in species richness and diversity among different treatment groups.

In the results of intestinal microbiota diversity analysis, the OTU number of mice in each group was different. The intestinal microbiota diversity of the CK group was the highest, the YK group was second, the DH group was also relatively high, and the NK group was the lowest. These results indicated that a high-fat diet reduced the intestinal microbial diversity of mice, while the drug Xuezhikang and high-dose tea extract had a significant effect on the recovery of intestinal microbiota of mice. In the study of alpha and beta diversity, it was found that the CK group had the highest abundance and diversity of intestinal microbiota, the NK group had the lowest, and DL and DH groups had similar diversity of intestinal microbiota. The low and high doses for mice were set to be 5 and 20 times of the recommended amount for adults, respectively. The recommended amount of dried compound tea leaves for adults is 9 g/day. We usually put 3 g of dry tea in a tea pot and infuse it several times with boiling water, so it is equivalent to making three pots of the compound tea.

*Firmicutes* and *Bacteroidetes* are the two most abundant bacteria in the human intestine [31], which jointly promote host energy absorption and storage. The relative abundance of *Firmicutes* has a significant positive correlation with obesity, while *Bacteroidetes* has a significant negative correlation [32–34]. Generally, the ratio of *Firmicutes* to *Bacteroidetes* (F/B value) is used as an index to measure obesity, and the F/B value of obese individuals is higher than that of normal individuals [32], and current research suggests that the internal environment of intestinal microbiota is an important factor affecting obesity and fat accumulation [18]. This index is similar in human and mice. For example, researchers detect the effects of Pu-erh tea both in mice and humans and got consistent results [35]. This study found that *Firmicutes* was highest in the NK group and lowest in the DH group, while *Bacteroidetes* was lowest in the NK group, and the F/B value was highest, indicating that the high-fat diet caused intestinal microbial imbalance and that supplementation with tea was able to normalize that imbalance to a certain degree.

Intestinal microbiota are also closely related to host metabolism. Butyrate is the most important nutrient for intestinal cell renewal. The increase of butyrate induced by host genes can improve insulin response and reduce the risk of type 2 diabetes [36]. In this study, the trend of relative abundance of *Lachnospiraceae* was the same as similar studies. In addition, the composition of the intestinal microbiota in patients with spondyloarthropathy is related to the decrease in *Dorea* in the genus *Trichophyton* [37]. There is a characteristic bacterial interaction network in intestinal microorganisms. In this study, *Ruminococcus* was associated with metabolic disorders, and its relative abundance in intestinal tract of obese hosts was high [38, 39]. Relevant studies have shown that the disorder of interaction network between *trichospirillaceae* and Ruminococcaceae can cause Crohn's disease (CD) and ulcerative colitis (UC), while adhesive invasive *Escherichia coli* (AIEC) can utilize the inflammation caused by *Salmonella* and colonize stably in the intestine, and AIEC colonization can promote colon fibrosis [40, 41]. Vibrio desulfuricans (*Desulfovibrio*) is an intestinal pathogenic bacterium that can produce endotoxin. The increase of *Proteobacteria* will cause intestinal immune disorders and lead to intestinal inflammation, which is consistent with the results of the detection of dominant bacteria in mice intestinal microbiota induced by high-fat diet in this study. It was rarely or not found in other groups.

To sum up, compound tea could increase the abundance and diversity of intestinal microbiota in mice, which had a positive effect on the regulation of intestinal microbiota disorder caused by high-fat diet. Compound tea increased the number of beneficial microbiota to a certain extent and inhibited the growth of the relevant pathogenic bacteria. These results provide useful information for the treatment of hyperlipidemia.

## Figures and Tables

**Table 1:** Statistics of sample data and OTU numbers.

Sample	Raw reads	Final reads	OTUs
CK1-FB	41105	31931	197
CK2-FB	53507	43568	214
CK3-FB	66881	51034	223
DH1-FB	56185	42320	187
DH2-FB	59501	46491	192
DH3-FB	59414	49647	189
DL1-FB	43549	39312	186
DL2-FB	75001	61896	184
DL3-FB	76483	65921	180
NK1-FB	61254	49908	169
NK2-FB	52906	44321	188
NK3-FB	60807	55289	189
YK1-FB	50510	42059	180
YK2-FB	76126	62163	201
YK3-FB	78286	61479	199

## Data Availability

The data that support the findings of this study are openly available in the GenBank of NCBI at https://www.ncbi.nlm.nih.gov, reference number: PRJNA633980.

## Supplementary Materials

Supplementary Materials Table S1: extract compounds from tea leaves. Table S2: body weight of mice before and after treatment. Figure S1: structure of the microbial and abundance changes at class level. Figure S2: structure of the microbial and abundance changes at family level. Figure S3: structure of the microbial and abundance changes at order level. Figure S4: structure of the microbial and abundance changes at genus level. Figure S5: structure of the microbial and abundance changes at species level. Figure S6: cladogram from LEfSE analysis.
