# Supplementary material for: Effect of Duyun Compound Green Tea on Gut Microbiota Diversity in High-Fat-Diet-Induced Mice Revealed by Illumina High-Throughput Sequencing
Source: Evid Based Complement Alternat Med. 2021 Feb 9;2021:8832554. doi: 10.1155/2021/8832554 (PMC7886516; doi:10.1155/2021/8832554)
Supplement: Supplementary Materials — Table S1: extract compounds from tea leaves. Table S2: body weight of mice before and after treatment. Figure S1: structure of the microbial and abundance changes at class level. Figure S2: structure of the microbial and abundance changes at family level. Figure S3: structure of the microbial and abundance changes at order level. Figure S4: structure of the microbial and abundance changes at genus level. Figure S5: structure of the microbial and abundance changes at species level. Figure S6: cladogram from LEfSE analysis. [file 8832554.f1.docx]

**Table S1** Extract compounds from tea leaves

| Chemicals | Content (%) |
| --- | --- |
| L-EGC | 1.7 |
| D.L-GC | 1.4 |
| L-EC | 2.5 |
| L-EGCG | 5.8 |
| L-ECG | 2.3 |
| Total catechins | 13.7 |
| Total polyphenols | 24.1 |
| Amino acids | 4.2 |
| Caffeine | 4.4 |
| Soluble saccharides | 3.1 |

**Table S2** Body weight of mice before and after treatment

| Group | Initial weight (g) | Final weight (g) |
| --- | --- | --- |
| CK | 42.3±1.4 Aa * | 45.9±1.0 De |
| NK | 42.9±1.1 Aa | 54.1±1.6 Aa |
| YK | 42.5±1.8 Aa | 47.8±1.0 Ccd |
| DL | 42.5±1.7 Aa | 50.6±1.5 Bb |
| DH | 42.1±0.7 Aa | 46.5±1.2 CDde |

* Different letters indicate significant difference among groups (Uppercase: *p* < 0.01, Lowercase: *p* < 0.05).


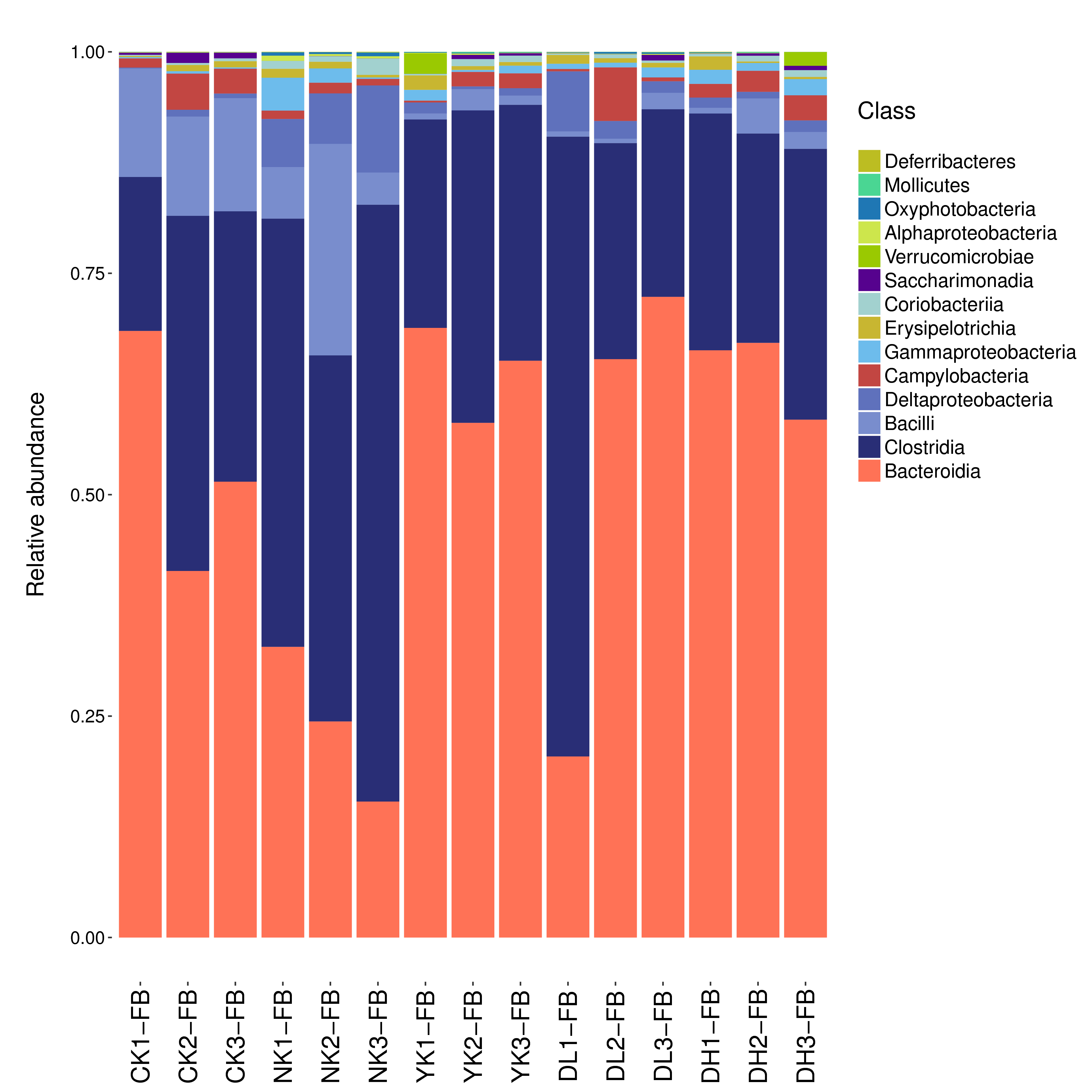


**Fig. S1** Structure of the microbial and abundance changes at class level.


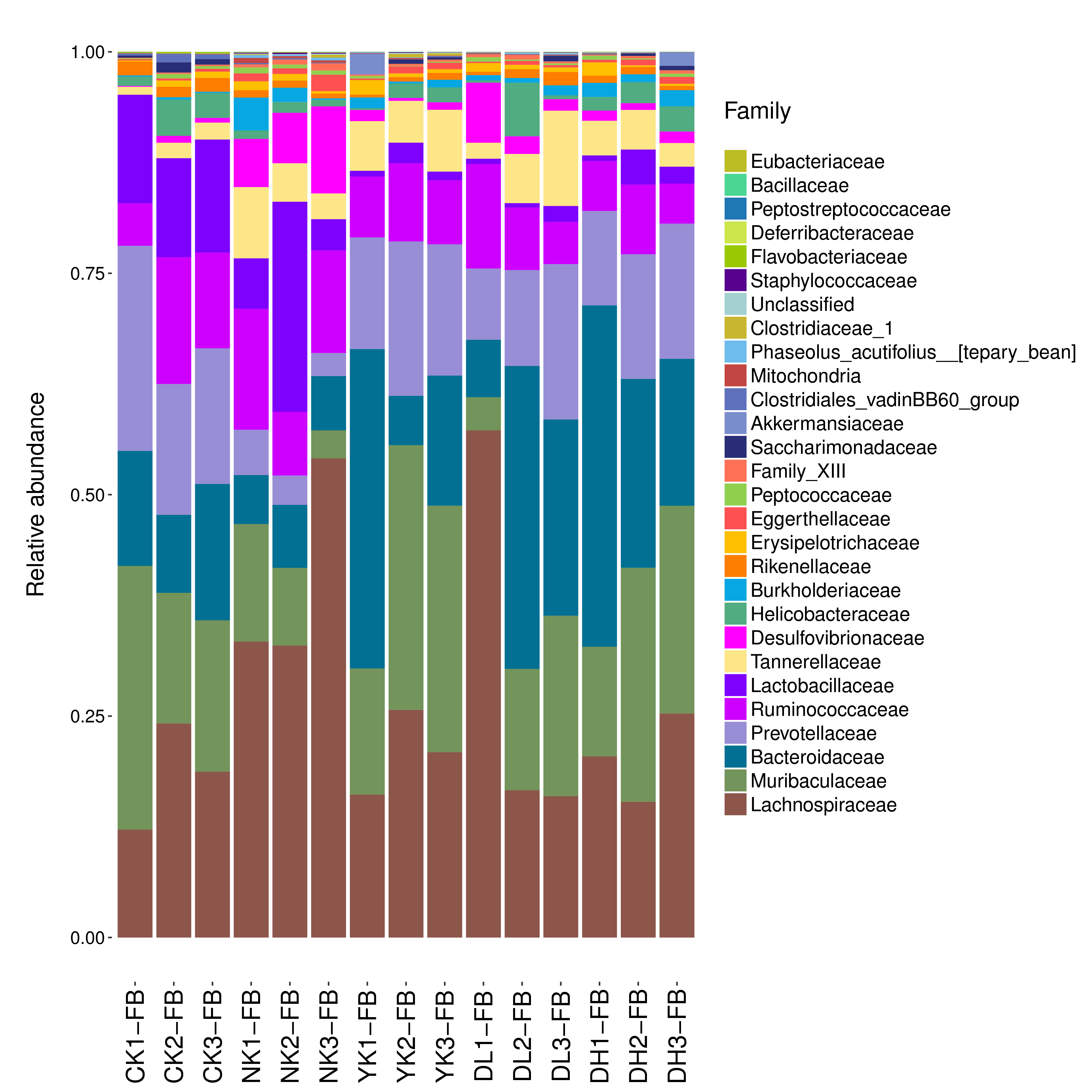


**Fig. S2** Structure of the microbial and abundance changes at family level.


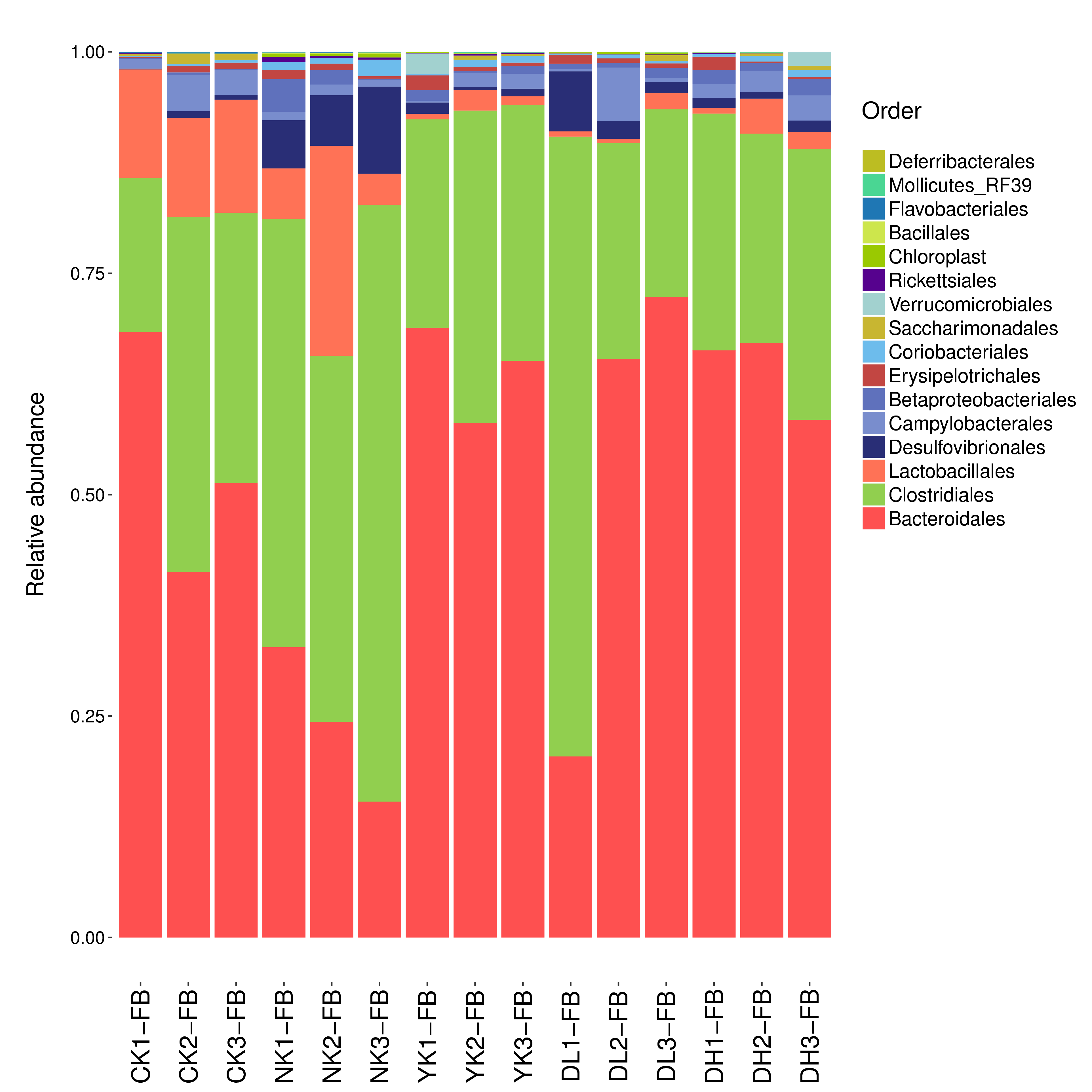


**Fig. S3** Structure of the microbial and abundance changes at order level.


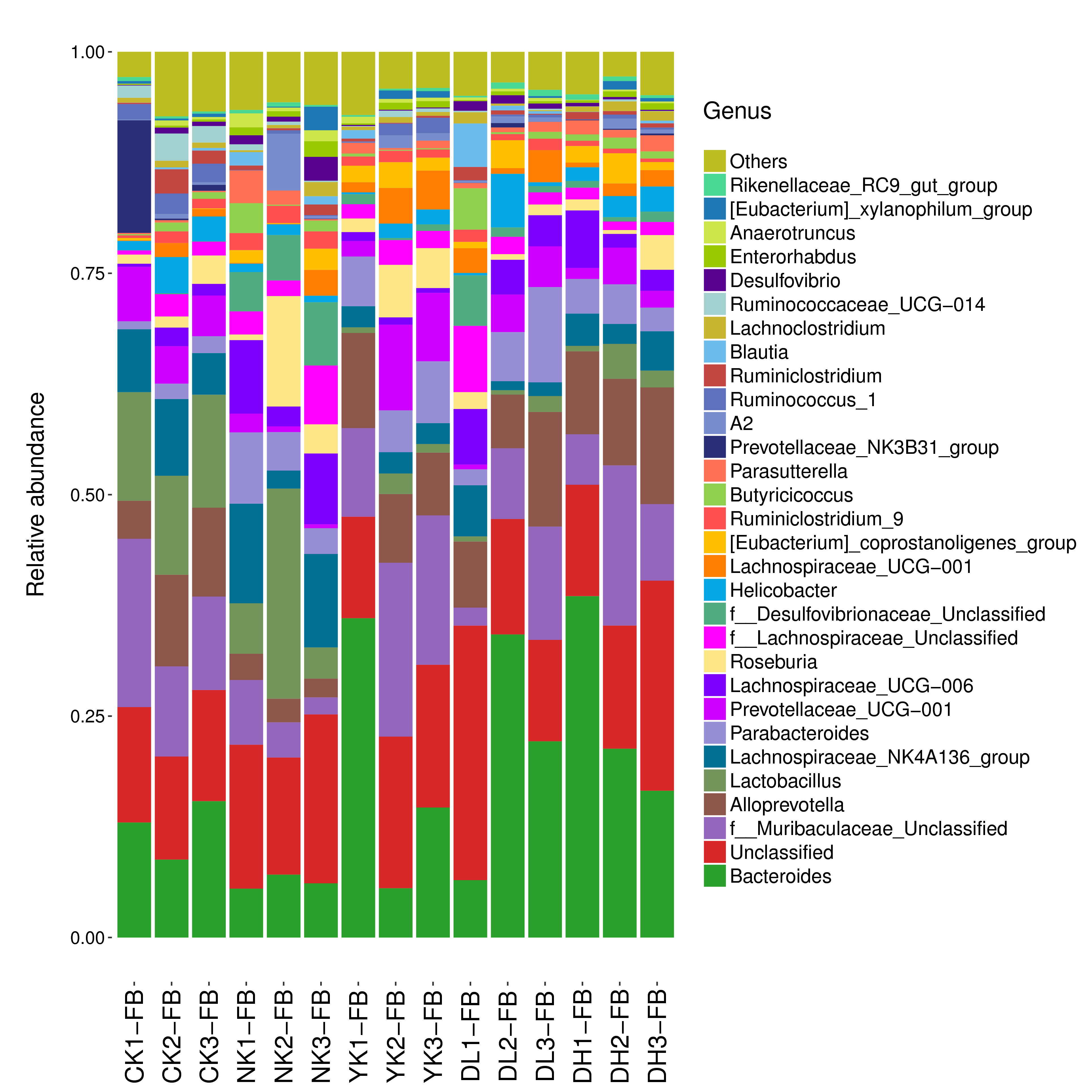


**Fig. S4** Structure of the microbial and abundance changes at genus level.


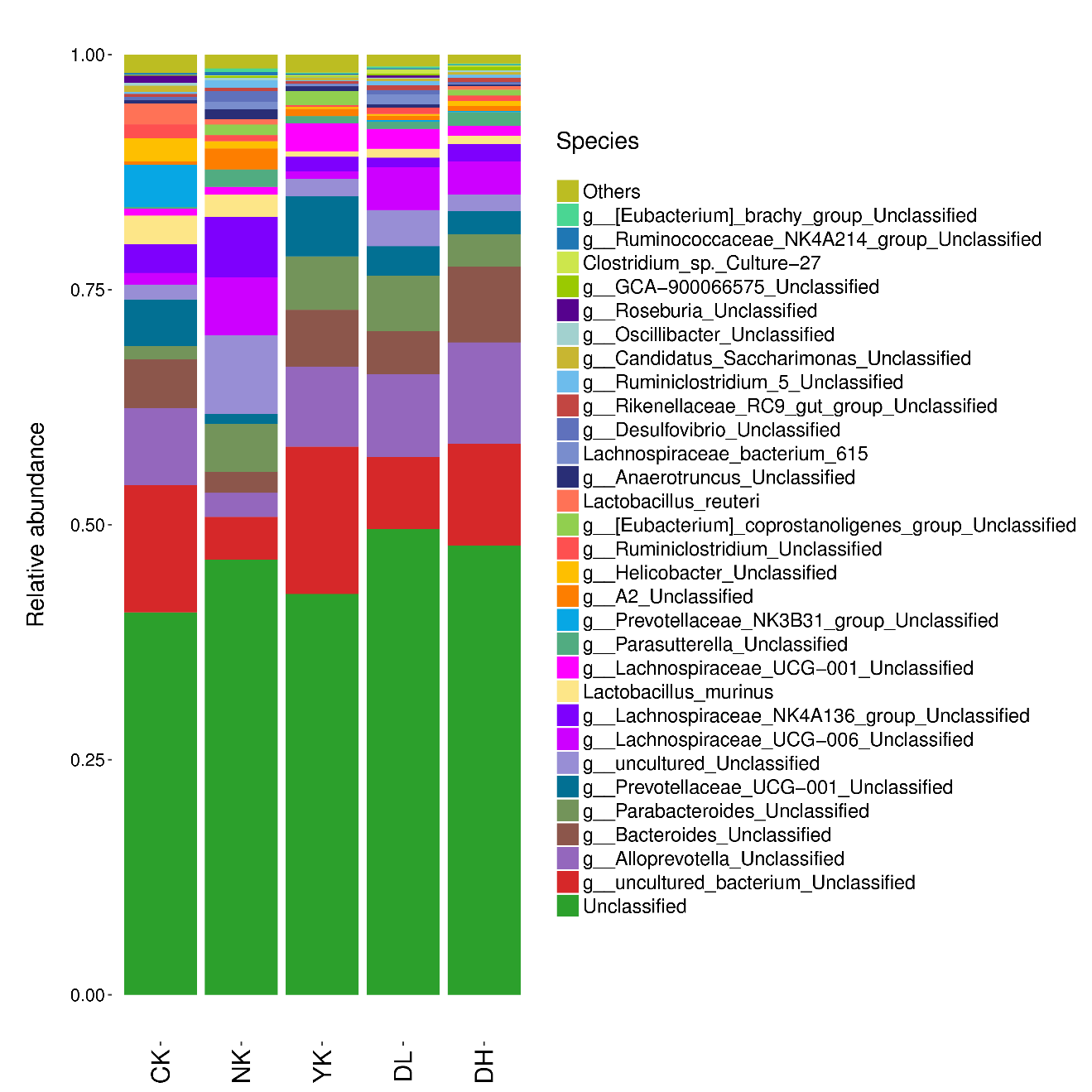


**Fig. S5** Structure of the microbial and abundance changes at species level.


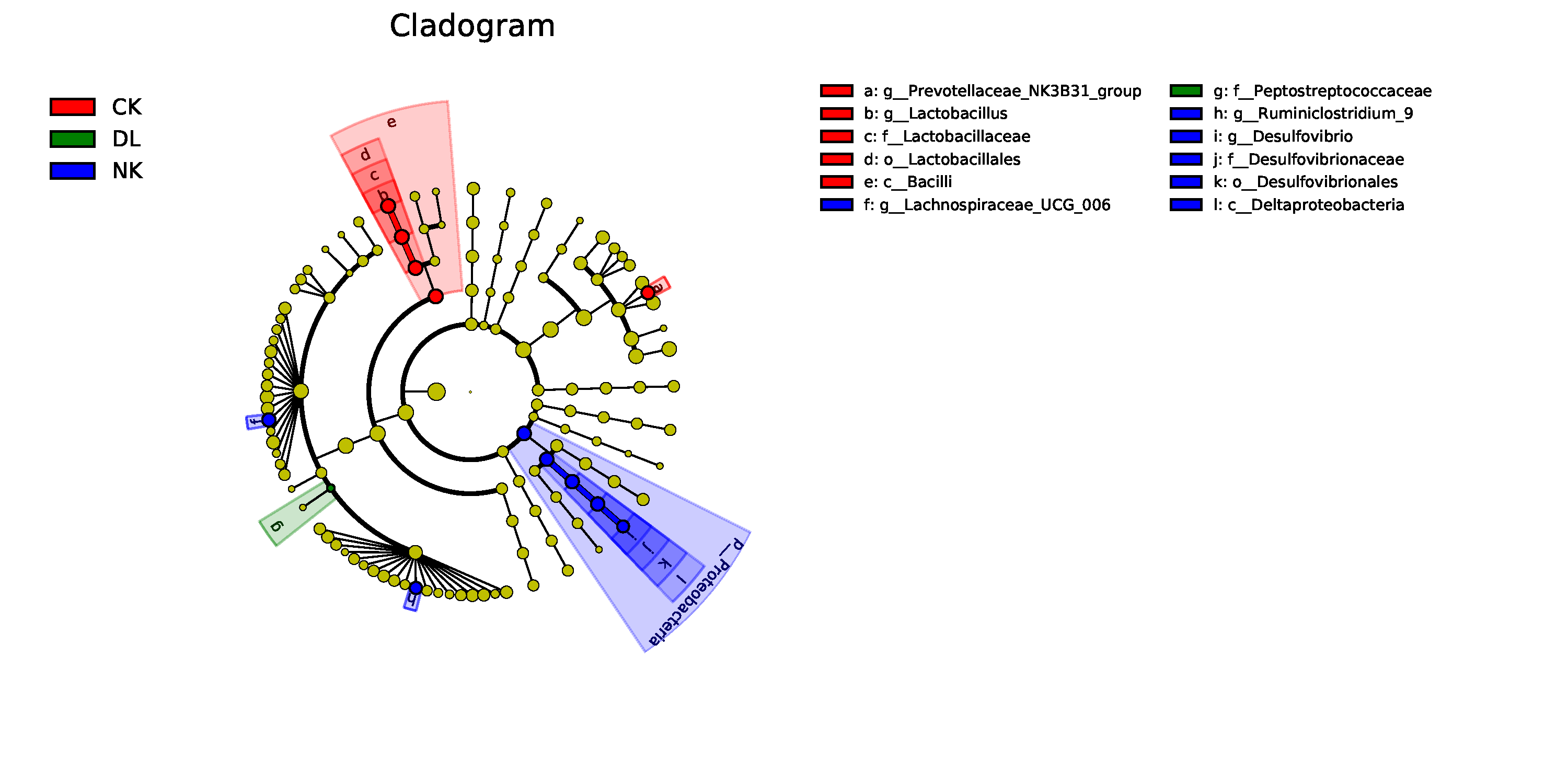


**Fig. S6** Cladogram from LEfSE analysis.
